# Breastfeeding dynamically changes endogenous oxytocin levels and emotion recognition in mothers

**DOI:** 10.1098/rsbl.2020.0139

**Published:** 2020-06-03

**Authors:** Michiko Matsunaga, Takefumi Kikusui, Kazutaka Mogi, Miho Nagasawa, Rumi Ooyama, Masako Myowa

**Affiliations:** 1 Department of Education, Kyoto University, Yoshida-honmachi, Sakyo-ku, Kyoto 606-8501, Japan; 2 School of Veterinary Medicine, Azabu University, Veterinary Medicine, 1-17-71 Fuchinobe, Chuo-ku, Sagamihara-shi, Kanagawa 252-5201, Japan

**Keywords:** breastfeeding, mothers, endogenous oxytocin, emotion recognition, negative buffering

## Abstract

Breastfeeding behaviours can significantly change mothers' physiological and psychological states. The hormone oxytocin may mediate breastfeeding and mothers' emotion recognition. This study examined the effects of endogenous oxytocin fluctuation via breastfeeding on emotion recognition in 51 primiparous mothers. Saliva oxytocin was assessed before and after the manipulation (breastfeeding or holding an infant), and emotion recognition tasks were conducted. Among mothers who breastfed daily, mothers with more increased levels of oxytocin after breastfeeding showed more reduced negative recognition and enhanced positive recognition of adult facial expressions. These oxytocin functions accompanying breastfeeding may support continued nurturing behaviours and also affect the general social cognition of other adults beyond any specific effect on infants.

## 1. Introduction

Caregivers need to adjust their parenting behaviours by inferring infants' physiological and emotional states from nonverbal cues (e.g. facial expressions). The maternal nurturing experience can in fact influence mothers' ability to recognize not only their infants' facial expressions but also adults' [1]. As part of the nurturing experience, breastfeeding is an important factor that could lead to plastic changes in mothers' psychological processing. Previous studies have shown that breastfeeding buffers psychological stress and anxiety [2–4] and enhances positive feelings when mothers see their infants' facial expressions [5]. Similarly, a recent study showed that long-lasting breastfeeding is related to perceptual reduction of negative facial expressions and sensitive identification of positive facial expressions [6].

The oxytocin system is a compelling mechanism related to both lactation and psychological processing in mothers. Oxytocin is a neuropeptide hormone synthesized in the paraventricular and supraoptic nuclei of the hypothalamus by infants' suckling [7]. Peripheral endogenous oxytocin concentration during breastfeeding can be measured using saliva and plasma [8–11]. Further, over the past two decades, many studies have shown by administering intranasal oxytocin to male study participants that oxytocin can affect social cognition and behaviours. Previous studies have shown that exogenous oxytocin buffers negative emotional processing, such as perceived stress and anxiety [12,13] and recognition of negative facial expressions [14,15]. Furthermore, exogenous oxytocin also seems to enhance recognition of positive facial expressions [16]. However, previous findings examining exogenous oxytocin's effect on emotion recognition are sometimes inconsistent. Several studies have shown that intranasal oxytocin does not influence the recognition of emotional facial expressions such as happiness and anger [17,18]. One of the possible reasons for the inconsistent findings is that the individual differences in endogenous oxytocin (levels of oxytocin in the body) concentrations and fluctuations have been ignored within the oxytocin-administration literature [19].

Considering the female endogenous oxytocin system, it is also necessary to control for the menstrual cycle [20]. Specifically, short-term (tonic) and long-term (phasic) breastfeeding are factors that greatly influence a mother's endogenous oxytocin concentration and fluctuation. For example, peripheral oxytocin concentrations are higher in breast-feeders than formula-feeders [11], and breastfeeding behaviour itself causes dynamic fluctuations in the mother's oxytocin levels [8]. However, previous studies only compared breastfeeding and formula-feeding mothers and did not focus on the individual differences of oxytocin across mothers via breastfeeding. To further understand how oxytocin impacts socio-emotional processing in mothers, it is necessary to consider the impact of both tonic and phasic breastfeeding experiences on endogenous oxytocin.

This study aimed to reveal whether maternal oxytocin fluctuation was related to mothers' emotion recognition by focusing on individual differences in oxytocin fluctuation associated with breastfeeding. To do so, we conducted two kinds of emotion recognition tasks. We assessed mothers' reaction time, accuracy and arousal rating when detecting or identifying emotional facial expressions of other adults. To differentiate the effects of tonic and phasic breastfeeding experiences on mothers' oxytocin fluctuation, we determined whether the duration and frequency of mothers' breastfeeding experience could influence their oxytocin concentration before and after breastfeeding as a tonic effect, and we compared two kinds of infant manipulation (breastfeeding and holding an infant) to compare the oxytocin changes as a phasic effect.

## 2. Methods

**Figure 1.**
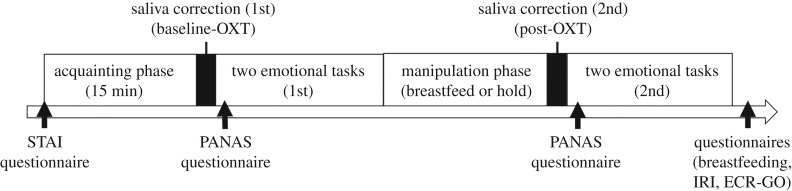
The experimental procedure. OXT, oxytocin; STAI, State-Trait Anxiety Inventory; PANAS, Positive and Negative Affect Schedule; IRI, Interpersonal Reactivity Index; ECR-GO, generalized version of the Experience in Close Relationships Inventory.

We analysed 51 primiparous mothers' data. All participants continued breastfeeding their 2- to 9-month-old infants during the study period. None reported currently having any psychiatric disorders or taking any medication (see the electronic supplementary material for more information about sample size and participants' characteristics, S1, S2). Figure 1 shows the experimental procedure. After we had gained informed consent, we provided a 15 min get-acquainted period during which mothers did not touch their infants. Then, we collected saliva samples, conducted a questionnaire about affect, and conducted two emotion recognition tasks twice, both before and after the manipulation. During the manipulation phase, participants either breastfed or held their infants for 15 min. We randomly assigned our participants to either of the two conditions (breastfeeding or holding) and informed them of the same in advance. Finally, the mothers completed all the remaining questionnaires, as described below. Since mothers conducted the experiment alone in the soundproofed room, fathers or grandmothers of the infants also joined the experiment to care for the infants along with our research assistant.

## 3. Measurements

### (a) Questionnaires

We obtained information about breastfeeding duration and the mean frequency of current breastfeeding as tonic breastfeeding indices through an in-house developed questionnaire. The breastfeeding duration is the number of days of continuous breastfeeding at the time of the experiment. Additionally, four kinds of questionnaires were administered to investigate the potential effects of maternal characteristics (i.e. age and years of education), maternal affect, anxiety, empathy concern and attachment style on individual differences in oxytocin (see the electronic supplementary material, S2).

### (b) Saliva oxytocin

Prior to saliva collection, mothers drank a glass of water to rinse out their mouths. After 15 min, 1.5–2.0 µl of saliva were collected by having mothers expectorate down a straw into a cryovial using the Saliva Collection Aid (Salimetrics). The samples were stored at −80°C until assayed. Saliva oxytocin concentrations were measured using a commercial ELISA kit (ADI-901–153A – Enzo Life Sciences), following the manufacturer's protocol. The plate was read at an optical density of 450 nm using a microplate reader. The intra-assay coefficient of variation was 1.77–2.01% (see the electronic supplementary material, S3).

### (c) Emotion recognition tasks

We conducted two emotion recognition tasks. One was an emotion detection task using greyscale images of adults' neutral, angry and happy facial expressions. Each stimulus display consisted of eight face stimuli placed around the central fixation point (the task design was based on [14,21,22]). Participants detected as quickly and accurately as possible for each stimulus whether all the eight faces were the same or if one face showed a different emotion. The other task was emotion identification. The stimuli were morphed so that the facial expression changed gradually from neutral to a full-blown emotional expression (angry, fearful, happy or sad) over the course of 3000 ms (the task design was based on [6]). Participants identified an emotional category as quickly and as accurately as possible. Participants also evaluated the arousal intensity of the emotion using a nine-point scale ranging from 0 (low arousal) to 8 (high arousal). We analysed reaction time only for correct responses and considered the percentage of correct responses for determining the accuracy of each task (see the electronic supplementary material, S4).

### (d) Analysis

We calculated the amount of change in oxytocin and task performance (i.e. reaction time, accuracy and arousal rating) from the pre-manipulation baseline to post-manipulation by subtracting baseline scores from post scores. To investigate how individual differences in oxytocin fluctuation related to emotion recognition task performances, we performed multiple regression analysis. As a first step, we performed correlation analysis in order to investigate relationships between changes in oxytocin concentration (Δ-OXT) and all task performances (Δ reaction time, Δ accuracy, Δ arousal ratings). Then, we performed forced-entry multiple regression analysis to all significant correlations. As dependent variables, we entered task performances. As independent variables, we entered Δ-OXT and all possible variables related to the oxytocin in the prior analysis as regression predictors.

## 4. Results

### (a) Data preparation: tonic and phasic breastfeeding effect on oxytocin and possible factors

Correlation analysis revealed that the duration and frequency of tonic breastfeeding did not relate to any of the mothers' oxytocin (baseline-OXT, post-OXT, Δ-OXT). ANOVA (for baseline-OXT and post-OXT) and unpaired Student's t-test (for Δ-OXT) also revealed that mothers' oxytocin did not differ between phasic breastfeeding and holding groups (see the supplementary material, S5). However, correlation analysis revealed that post-OXT was significantly related to the post negative affect (*r*[27] = −0.45, *p* = 0.02) and anxiety tendency in attachment style (*r*[27] = −0.43, *p* = 0.03) in the breastfeeding group. Baseline-OXT, Δ-OXT in both groups and post-OXT in the holding group did not relate to any of the measures. Therefore, post negative affect and anxiety tendency in attachment style were included as regression predictors.

### (b) Relationships between oxytocin and emotion recognition task performances

**Figure 2.**
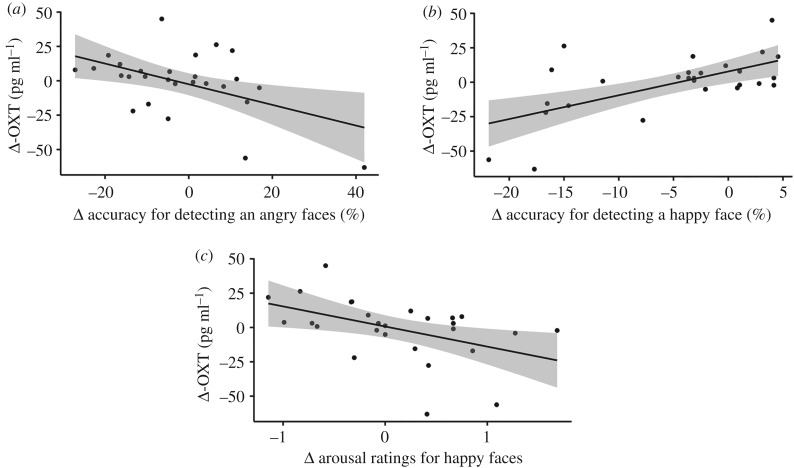
Significant relationships between Δ-OXT and Δ task performances in the breastfeeding group. Scatter plots and regression lines with 95% confidence intervals show the relationships between Δ-OXT concentration (pg/ml) (i.e. vertical axis) and (*a*) Δ accuracy for detecting an angry face from neutral faces (%), (*b*) Δ accuracy for detecting a happy face from angry faces (%), (*c*) Δ arousal rating for a happy face (i.e. horizontal axis).

First, correlation analysis revealed that Δ-OXT related to Δ accuracy for detecting an angry face from neutral faces (*r*[27] = −0.41, *p* = 0.04), Δ accuracy for detecting a happy face from angry faces (*r*[27] = 0.44, *p* = 0.02) and Δ arousal rating for a happy face (*r*[27] = −0.54, *p* = 0.004) only in the breastfeeding group (figure 2). With this information, we conducted forced-entry multiple regression analysis. Δ-OXT was the strongest predictor for all three task performances (table 1). This indicated that the more oxytocin increased, the more accuracy decreased for detecting an angry face, the more accuracy increased for detecting a happy face and the more arousal rating decreased for happy faces. Δ-OXT in the holding group did not relate to any of the task performances; therefore, we did not conduct multiple regression analysis. Note that all *p*-values reported here are uncorrected.

**Table 1.** Results of multiple regression analysis which Δ-OXT predicted Δ emotion task performances.

	Δ accuracy for detecting an angry face from neutral faces			
predictor	*β*	s.e.	*t*-value	*p*
(constant)	6.84	9.74	0.70	0.49
post negative affect	0.04	0.73	0.22	0.83
anxiety tendency in attachment	−0.26	0.15	−1.40	0.18
Δ-OXT (pg/ml)	−0.51	0.12	−2.88	0.009
total model results	*adjusted R^2^* = 0.21*			
	*F*_3,26_ = 3.34			

	Δ accuracy for detecting a happy face from angry faces			
predictor	*β*	s.e.	*t*-value	*p*
(constant)	−3.07	4.79	−0.64	0.53
post negative affect	−0.23	0.36	−1.41	0.17
anxiety tendency in attachment	0.17	0.07	1.03	0.31
Δ-OXT (pg/ml)	0.65	0.06	4.13	0.0001
total model results	*adjusted R^2^* = 0.37**			
	*F*_3,26_ = 6.02			

	Δ arousal ratings for happy faces			
predictor	*β*	s.e.	*t*-value	*p*
(constant)	0.57	0.45	1.25	0.22
post negative affect	−0.39	0.03	−2.20	0.04
anxiety tendency in attachment	0.19	0.01	1.08	0.29
Δ-OXT (pg/ml)	−0.41	0.01	−2.43	0.02
total model results	*adjusted R^2^* = 0.27*			
	*F*_3,26_ = 4.19			

*p < 0.05, **p < 0.01.

*β*, standardized regression coefficient; s.e., standard error.

## 5. Discussion

This study investigated whether mothers' endogenous oxytocin fluctuations via breastfeeding affected emotion recognition. We found that increasing oxytocin through breastfeeding predicted decreasing accuracy of detecting negative (angry) facial expressions and increasing accuracy of detecting positive (happy) facial expressions. Consistent with our hypothesis, higher endogenous oxytocin fluctuations via short-term breastfeeding (i.e. before and after breastfeeding) buffered negative emotion recognition and enhanced positive emotion recognition.

Breastfeeding can reduce psychological stress, anxiety and negative facial expression recognition [2,6] and enhance positive feelings towards the infant and emotion recognition of happy adult facial expressions [5,6]. Our result showed that individual difference in oxytocin fluctuations predicted these psychological and perceptual changes in mothers. Reducing their sensitive reaction to negative emotional signals could allow mothers to continue breastfeeding by decreasing their psychological stress.

It is important to note that we used adult facial expressions as the stimuli. Our results therefore demonstrate that oxytocin fluctuation via breastfeeding could impact not only the specific relationship between mother and infant but also more general emotional processing towards other adults. This contention is supported by neuroimaging studies, which found that women's brains, from pregnancy until at least 2 years after birth, undergo plastic changes involved in the areas serving recognition other people's emotions [23], and intranasal oxytocin in mothers promotes sensitive neural responses to both infant and adult facial expressions [24].

Moreover, we found that more increased oxytocin owing to breastfeeding decreased the subjective rating of arousal for happy facial expressions. Previous studies revealed that intranasal oxytocin decreased amygdala activity [25,26]. Moreover, higher arousal stimuli, including positive emotional stimuli, generally relate to higher amygdala activity [27]. In fact, a study showed that postpartum mothers who were administered intranasal oxytocin had decreased amygdala activity and assigned low arousal ratings to negatively arousing stimuli [28]. Therefore, as one of the possibilities, increased oxytocin via breastfeeding is related to a lower arousal rating for happy facial expressions by mediating lower amygdala activity.

In our study, only oxytocin fluctuation before and after breastfeeding predicted changes in maternal emotion recognition, although there was no significant difference in oxytocin concentrations and fluctuations between the two groups (breastfeeding versus holding). Our data did not provide a direct explanation for this; however, one possibility is that breastfeeding, as opposed to holding an infant, is a multiplex behaviour including a variety of physiological effects such as hormones (e.g. prolactin) and HPA axis (i.e. stress) responses [29]. In particular, sucking has been shown to suppress HPA axis responses. A previous study showed that the increase in cortisol was more suppressed in the nursing group than in the holding group, and there was also a difference in subjective mood changes: calmness increased in the breastfeeding group but tension increased in holding group [29]. Intranasal oxytocin studies also showed that oxytocin suppressed HPA axis responses to stressors [12,30]. Therefore, it is possible that fluctuations in oxytocin hormones via breastfeeding affect emotional cognition in concert with other physiological systems.

In this study, individual differences in oxytocin levels and fluctuations were not associated with tonic and phasic breastfeeding experience. Although previous breastfeeding and oxytocin studies have compared exclusive breastfeeding mothers to formula feeding mothers [19,8,9], our findings suggested that individual differences in oxytocin were larger than breastfeeding or holding group differences when comparing mothers who breastfed daily. This may be owing to physical differences such as DNA and the degree of methylation which plastically change by early childhood pregnancy or postpartum experiences [31,32], rather than length and frequency of breastfeeding.

This study had several limitations. First, we did not control for multiple comparisons and only reported uncorrected results as mentioned in §4 (see [6]). However, the effect size of our results in multiple comparisons and in multiple regression analysis was also sufficiently large according to the criteria [33]. Furthermore, we did not record the state of the infant during the experiment (e.g. breastfeeding or holding manipulation). Although mothers conducted the experiment alone without hearing their infants' voice or cry, it could impact the mothers' stress and oxytocin responses. Further research on examining individual differences in oxytocin levels with other factors, such as the methylation and stress responses (e.g. cortisol), may enable early detection of mothers who are less likely to obtain an oxytocin-mediated effect in order to provide support for their physical and mental health.

In conclusion, our study revealed that individual differences in oxytocin fluctuations via breastfeeding predicted greater reduction in negative recognition and more enhancement in positive recognition of adult facial expressions. These findings provide evidence that endogenous oxytocin fluctuation accompanying breastfeeding is one of the mechanisms of perceptual and psychological change in mothers.

## Supplementary Material

The supplementary materials for participant's characteristics, methods and results

## Data accessibility

Data available from the Dryad Digital Repository: https://datadryad.org/stash/share/d81dU6B1yWBuUHkF7OHI8VPhIb-bm1ECfgPF0x_80V0.
