# Supplementary material for: Breastfeeding dynamically changes endogenous oxytocin levels and emotion recognition in mothers
Source: Biol Lett. 2020 Jun 3;16(6):20200139. doi: 10.1098/rsbl.2020.0139 (PMC7336852; doi:10.1098/rsbl.2020.0139)
Supplement: The supplementary materials for participant's characteristics, methods and results [file rsbl20200139supp1.docx]

**Electronic Supplementary Material (ESM)**

**Breastfeeding dynamically changes endogenous oxytocin levels and emotion perception in mothers**

Michiko Matsunaga,^1^ Takefumi Kikusui,^2^ Kazutaka Mogi,^2^ Miho Nagasawa,^2^ Rumi Ooyama,^2^ Masako Myowa^1^

*Corresponding author

Michiko Matsunaga

[matsunaga.michiko.66w@st.kyoto-u.ac.jp](mailto:matsunaga.michiko.66w@st.kyoto-u.ac.jp)

**This file includes:**

- **Supplementary text (Supplement S1-S5)**
- **References**

**S1: Participants and sample size**

Fifty-six primiparous mothers (mean age = 33.31, ranging from 26 to 45 years; *SD* = 4.96 years) participated in this study. Their infants’ mean age was 6.22 months (29 boys and 27 girls; ranging from 2 to 9 months; *SD* = 1.49 months). A maximum sample size of 56 participants was determined a priori by using G*Power. Assuming that we were going to use the regression analysis, we set the number of predictors as three, the effect size as 0.15 (medium level), and the power as 0.80.

Of the original 56 participants, we excluded five participants’ data because of the following reasons: incomplete questionnaire (N = 1); quitting breastfeeding (N = 2); extremely high oxytocin concentration and fluctuation, > 3 SD (N = 2). We analyzed the remaining 51 participants’ data.

**S2: Characteristics of participants and *Questionnaires***

Of the 51 participants, 37.25% of mothers continued exclusive breastfeeding, 27.45% of mothers supplemented with formula during the first few months but quit formula feeding and continued breastfeeding while they participated the experiment, and 35.29% mothers continued both to breastfeed and to supplement with formula. To control for the menstrual cycle, nine participants conducted their experiment during their luteal phase since they had resumed menstruation.

Four kinds of questionnaires were administered to investigate the potential effects of maternal affect, empathy concern, and attachment style on both accumulated breastfeeding experiences and oxytocin. First, we assessed anxiety using the Japanese version of the State-Trait Anxiety Inventory (STAI) [1, 2]. It can separately assess two aspects of anxiety: trait anxiety, which measures how one generally feels, and state anxiety, which measures how one feels at that moment. A higher total score indicates a high tendency toward anxiety. Second, we used the Positive and Negative Affect Schedule (PANAS), a frequently used measure of affect in adults [3]. PANAS contains two subscales: positive affect (e.g., excited, happy) and negative affect (e.g., nervous, frightened). Third, we used the Interpersonal Reactivity Index (IRI) [4], which is also a broadly used measure of empathy in adults. The IRI is composed of four dimensions of empathy: “perspective taking,” the tendency to take the perspective of others; “empathic concern,” the tendency to experience feelings of sympathy and compassion for those less fortunate; “personal distress,” the tendency to experience feelings of distress in response to discomfort in others; and “fantasy scale,” the tendency to transpose oneself into fictional situations such as novels or movies. Fourth, we used the Experience in Close Relationships Inventory (ECR) [5], a commonly used multidimensional scale for measuring attachment style in adults. We used the generalized version of the ECR developed in Japan (ECR-GO). Its reliability and validity have been confirmed [6]. It comprises two dimensions of attachment: anxiety tendency and avoidance tendency. Table S1 shows the characteristics of the participants including scores for oxytocin, accumulated breastfeeding experience, and each questionnaire. We also confirmed the group differences of all these factors; however, there were no significant differences.

Table S1.

*Summary of the Questionnaire and Oxytocin Data*

**S3: Saliva oxytocin**

Salivary oxytocin was measured by a commercially available kit (ENZO, ENZ-ADI900153A0001), following the manufacturer’s protocol. We used a speedvac evaporator at room temperature for 3 hours to dry 240 μL of saliva and reconstituted it with a 240 μL assay buffer. The 100 μL of liquid was used for the assay. In the dilution test, one sample was diluted in three steps, and estimated and assayed OT concentrations were compared. As result, there was a significant correlation between assayed and estimated concentrations (slope = 1.46, *R^2^* = 1.00) (Figure S2a). In the recovery test, there was also a significant correlation between assayed and estimated concentrations (slope = 0.43, *R^2^*=0.96) (Figure S2b).

Figure S2a. The dilution test for saliva OT assay. There was a significant correlation between the assayed concentrations and estimated concentrations.

Figure S2b. The recovery test for saliva OT assay. There was a significant correlation between the assayed concentrations and estimated concentrations.

**S4: Emotion recognition tasks**

**Emotion Detection Task**

***Apparatus and stimuli.***

The task design was based on previous studies [7, 8, 9]. Colored photographs of faces – four models (two females and two males) showing three facial expressions (neutral, angry, happy) – were chosen from the Kokoro Research Center’s (KRC) facial expression database [10]. These were modified into grayscale using Adobe Photoshop. The mean luminance of all images was aligned to the same level using MATLAB version R2017a (MathWorks). Each stimulus display consisted of eight face stimuli placed around the central fixation point (Supplemental Figure 3a). Each face had a vertical visual angle of 2.8 degrees and a horizontal visual angle of 2.0 degrees. The distance from the central fixation to the center of each face was 5.0 degrees of visual angle at a viewing distance of approximately 90 cm. There were seven types of display. The three kinds of *same* stimuli consisted of eight faces displaying the same expression (all-angry, all-happy, all-neutral). The four kinds of *discrepant* stimuli consisted of seven faces expressing the same emotion and one face expressing a discrepant emotion (1 angry - 7 neutral, 1 angry - 7 happy, 1 happy - 7 neutral, and 1 happy - 7 angry). For all these discrepant stimuli, the appearance location of the discrepant face (either angry or happy) was equiprobable and random in each of the eight locations. Half of the models (one female and one male) participated in the pre-manipulation task, and the others participated in the post-manipulation task. The combination of the model was randomly chosen in every experiment.

***Emotion detection task procedure***

Participants responded by using a numeric keypad. They were told to press one of two keys using their right and left index fingers (either “4” or “6”). If all the faces were “the same,” they pressed one of the keys. If one face showed a “different” emotion from the other seven faces, they pressed the other key. The experimenter did not explicitly explain the procedure to participants by using the terms “angry” or “happy.” The two response keys (either “4” or “6”) were counterbalanced between the participants. They were encouraged to respond as quickly and as accurately as possible to each stimulus. Each trial started with a fixation cross (+) presented at the center of the computer screen for 500 ms. Next, the stimulus was presented, and it remained on the screen until the participant responded (Supplemental Figure 3a). In the pre-manipulation task, each participant underwent 35 practice trials (3 same and 32 discrepant displays) and 192 experimental trials. A break was inserted after every 48 trials. In the post-manipulation task, participants did not perform practice trials, but they performed almost the same number of trials as that in the pre-manipulation task. Each participant underwent a total of 224 trials in the post-manipulation task, with a break after every 56 trials.

***Analysis of the emotion detection task***

The mean reaction time of correct responses was calculated for each stimulus and for each participant, excluding measurements out of the total mean ± 2 SD as artefacts. Preliminary analysis was conducted for accuracy and errors. The error rates were slightly higher (> 10%) than those in previous studies, which only reported reaction times. Therefore, we also analyzed the percentage of correct responses as an accuracy index similar to Guastella et al. [7].

**
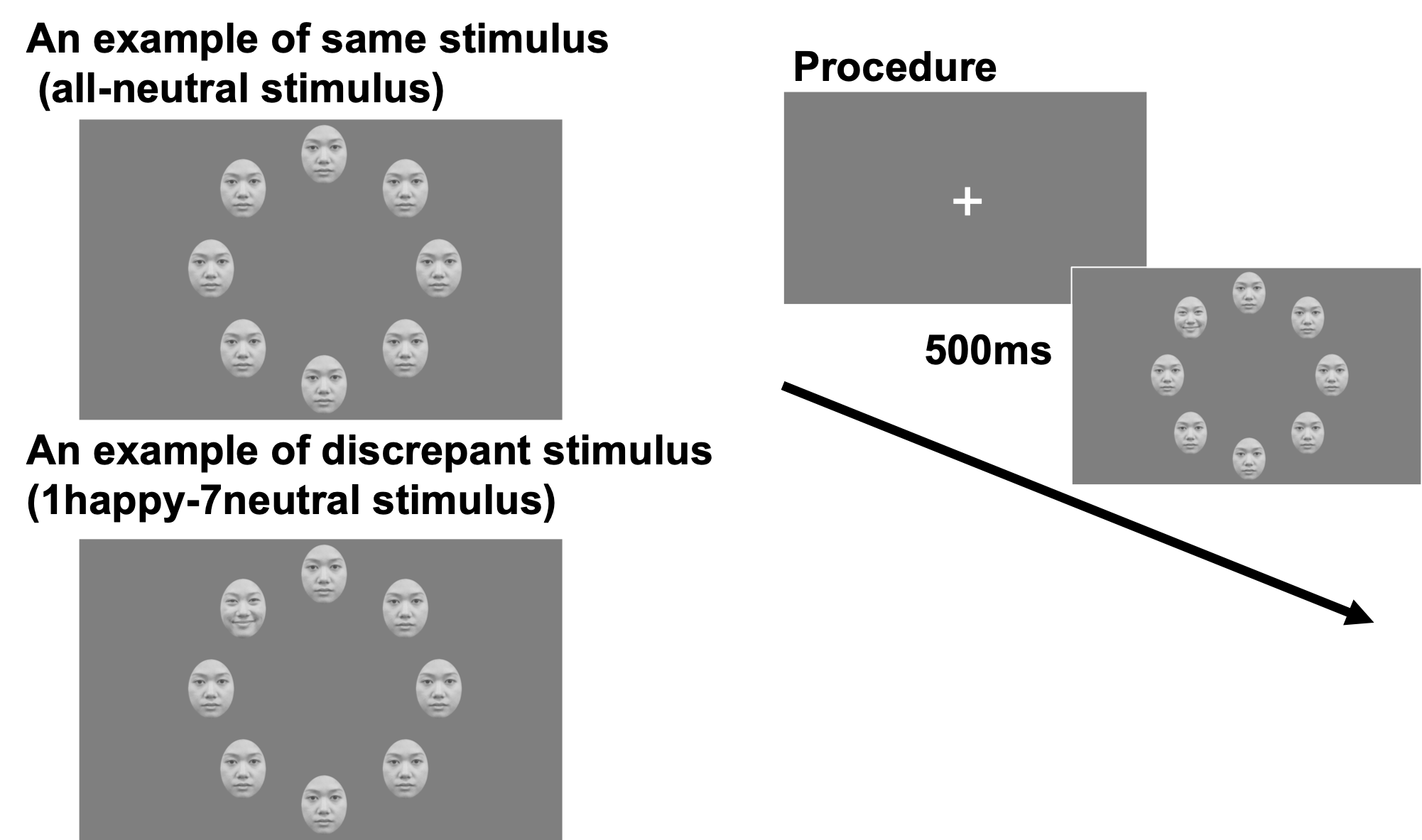
**

*Supplemental Figure 3a.* Stimuli and procedure of emotion detection task. The figures on the left are an example of the stimuli. Each trial started with a fixation cross (+) presented at the center of the computer screen for 500 ms. Then the stimulus was presented and remained on the screen until the participant responded.

**Emotion Identification Task**

***Stimuli***

The design of the emotion identification task was similar to that described in Krol et al. [11]. Images of 24 models (12 females and 12 males) showing five emotions (neutral, angry, fearful, happy, sad) were again taken from the KRC’s facial expression database. An additional female model’s facial expression was taken as a stimulus for practice trials. These 25 models were different from the four models used in the emotion detection task. The images (358 × 480 pixels) were morphed so that the facial stimuli changed gradually from neutral to a full-blown emotional expression (angry, fearful, happy, sad) over the course of 3000 ms. This resulted in a total of 100 morphed videos. For practice trials, each of the four emotional videos of one model was repeatedly presented three times in random order. Participants completed a total of 12 practice trials. As test trials, they completed 48 trials (12 models × 4 emotions). Half of the 24 models’ emotional videos were used in the pre-manipulation task, and the remaining 12 models’ videos were used in the post-manipulation task. The models presented in the pre- and post-manipulation tasks were randomly chosen in every experiment and presented in a random order.

***Emotion identification task procedure***

Each trial started with a fixation cross (+) presented at the center of the computer screen for 2000 ms. Next, a morphed movie was presented as a stimulus. Participants were instructed to press the key of the corresponding emotion with the thumb, index, middle, and ring fingers of their right hand as quickly and accurately as possible. Four labelled response keys corresponded to each emotion. The key correspondences were randomly changed for every participant. After identifying the emotional category, participants were asked to evaluate the arousal intensity of the emotion using a nine-point scale. We presented the question, “How strongly did you feel the negative or positive emotion?” as a prompt. Subsequently, a fixation circle (●) was presented for three to six seconds, a time window randomly selected by computer programming in every trial (Supplemental Figure 3b).

***Analysis of emotion identification task***

We analyzed reaction time, accuracy, and arousal intensity for each facial expression. Reaction times were analyzed for correct answers only. The percentage of correct responses was used as an accuracy index. The arousal intensity scores ranged from 0 (low arousal) to 8 (high arousal).

**
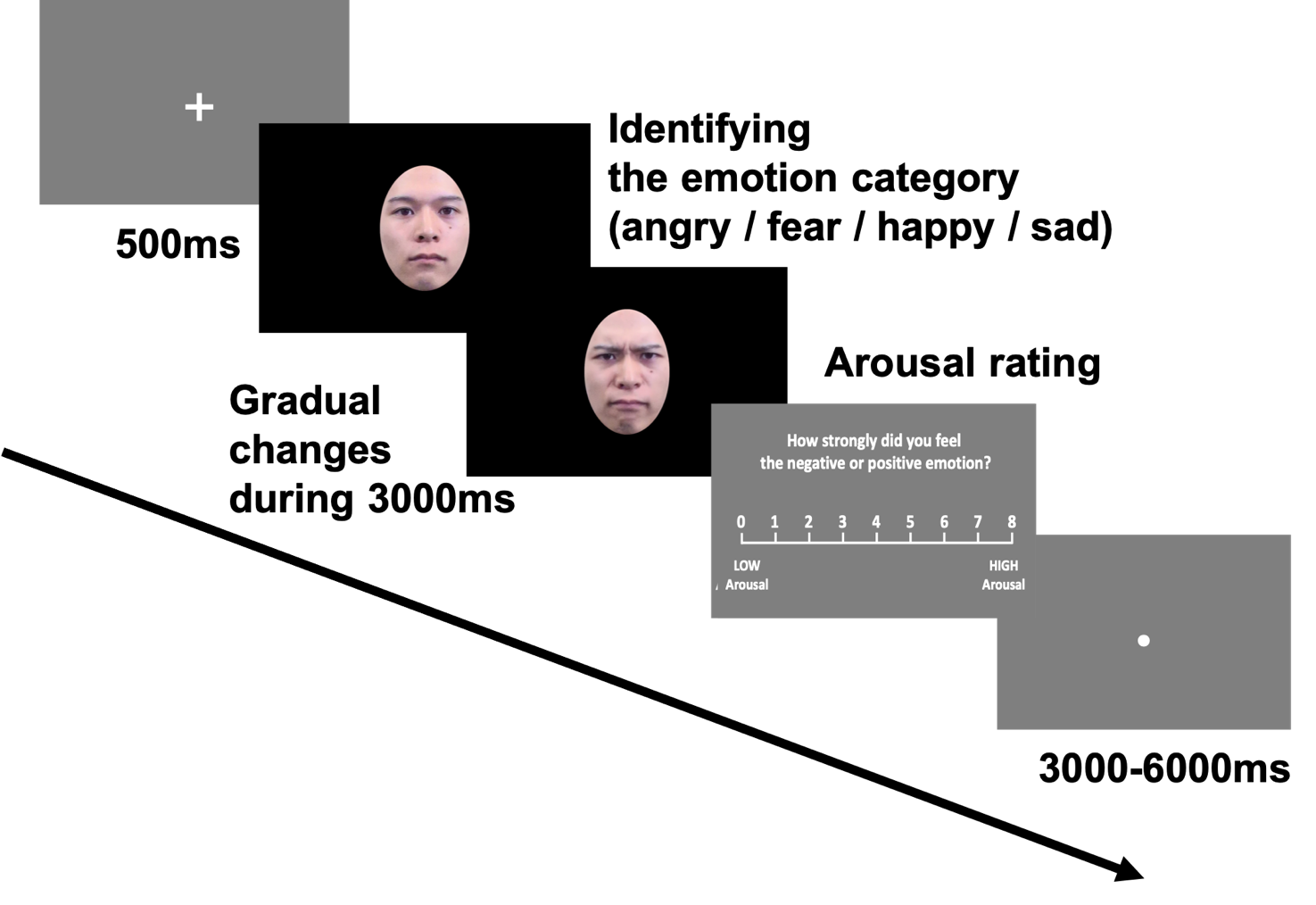
**

*Supplemental Figure 3b.* **E**motion identification task procedure. As the stimuli, morphed movies gradually changed from neutral to expressing an emotion during 3000 ms. Participants identified the emotion category and evaluated the arousal using a nine-point scale.

**S5: Results of *tonic and phasic breastfeeding effect on oxytocin***

For phasic breastfeeding effect on oxytocin, ANOVA analysis revealed that there was no significant group difference (*F*_1, 48_ = .18, *p* = .67, *η^2^* = .004), main effect of collection time (*F*_1, 48_ = .004, *p* = .95, *η^2^* = .000), along with an interaction between the group and the collection time (*F*_1, 48_ = .13, *p* = .72, *η^2^* = .003). An unpaired Student’s t-test for Δ OXT also showed that there was no significant difference between the breastfeeding and infant-holding groups (*t*_1,49_ = - .37, *p* = .72, *d* = .10).


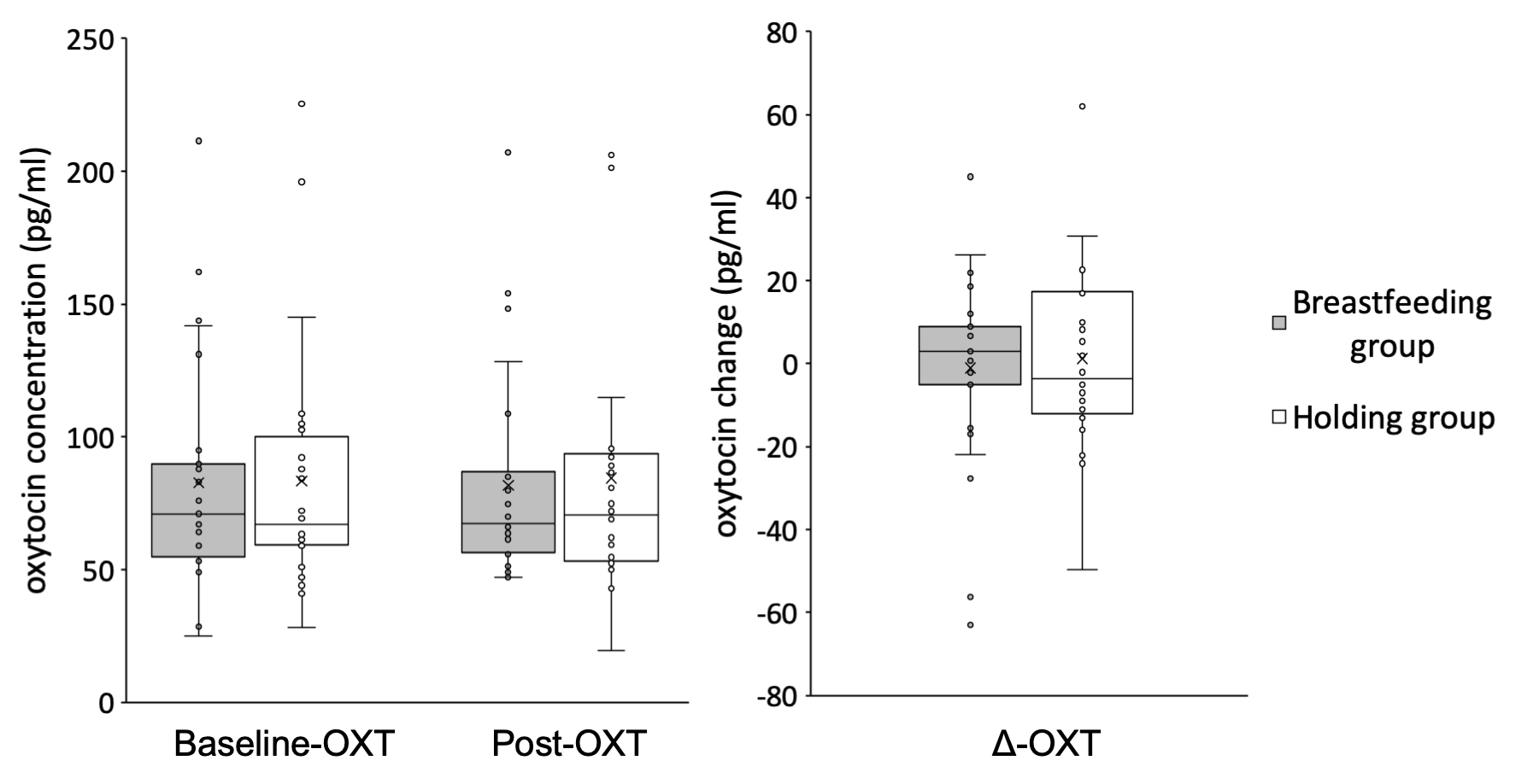


*Supplemental Figure 4*. Box plots show the difference in oxytocin between the breastfeeding and holding-infant groups. The vertical axis indicates oxytocin concentration (pg/ml). The circles plot the oxytocin concentration data of each participant.

**References**

1. Spielberger, C.D., Gorsuch, R.L. & Lushene, R.E. 1970 STAI manual for the State-Trait

Anxiety Inventory (“self-evaluation Questionnaire”). *Palo Alto, CA: Consulting Psychologists Press*.

1. Shimizu, H. & Imae, K. 1981 State-Trait Anxiety Inventory in Japanese for university students. *Educ Psychol*, **29**(4), 62-67.
2. Watson, D., Clark, L.A. & Tellegen, A. 1988 Development and validation of brief measures of positive and negative affect: The PANAS scales. *J Pers Soc Psychol*, **54**(6), 1063–1070.
3. Davis, M.H. 1983 Measuring individual differences in empathy: evidence for a multidimensional approach*. J Pers Soc Psychol*, **44**(1), 113–126.
4. Brennan, K.A., Clark, C.L. & Shaver, P.R. 1998 Self-report measurement of adult attachment: an integrative overview. In Simpson, A.J. & Rholes, W.S. (Eds.) *Attachment Theory and Close Relationships*, pp. 46–76. New York: The Guilford Press.
5. Nakao, T. & Kato, K. 2004. Examining reliabilities and validities of adult attachment scales for “the generalized other.” *Psychological Research of Kyushu University*, **5**, 19–27.
6. Guastella, A.J., Carson, D.S., Dadds, M.R., Mitchell, P.B., Cox, R.E. 2009 Does oxytocin influence the early detection of angry and happy faces? *Psychoneuroendocrino*, **34**(2), 220–225.
7. Fox, E., Lester, V., Russo, R., Bowles, R.J., Pichler, A. & Dutton, K. 2000 Facial expressions of emotion: are angry faces detected more efficiently? *Cognition and Emotion* **14**, 61-92. (doi:10.1080/026999300378996).
8. Sato, W. & Yoshikawa S. 2010 Detection of emotional facial expressions and anti- expressions. *Vis Cogn*, **18**(3), 369–388.
9. Ueda, Y., Nunoi, M., & Yoshikawa, S. (in press) Development and validation of the Kokoro Research Center (KRC) facial expression database. Psychologia, 61(3).
10. Krol, K.M., Kamboj, S.K., Curran, H.V. & Grossmann, T. 2014 Breastfeeding experience differentially impacts recognition of happiness and anger in mothers. *Sci Rep*, **4**, 7006.
